# CIEVaD: A Lightweight Workflow Collection for the Rapid and On-Demand Deployment of End-to-End Testing for Genomic Variant Detection

**DOI:** 10.3390/v16091444

**Published:** 2024-09-11

**Authors:** Thomas Krannich, Dimitri Ternovoj, Sofia Paraskevopoulou, Stephan Fuchs

**Affiliations:** Genome Competence Center, Robert Koch Institute, Nordufer 20, 13353 Berlin, Germany; krannicht@rki.de (T.K.);

**Keywords:** variant detection, genomics, testing, continuous integration, evaluation

## Abstract

The identification of genomic variants has become a routine task in the age of genome sequencing. In particular, small genomic variants of a single or few nucleotides are routinely investigated for their impact on an organism’s phenotype. Hence, the precise and robust detection of the variants’ exact genomic locations and changes in nucleotide composition is vital in many biological applications. Although a plethora of methods exist for the many key steps of variant detection, thoroughly testing the detection process and evaluating its results is still a cumbersome procedure. In this work, we present a collection of easy-to-apply and highly modifiable workflows to facilitate the generation of synthetic test data, as well as to evaluate the accordance of a user-provided set of variants with the test data. The workflows are implemented in Nextflow and are open-source and freely available on Github under the GPL-3.0 license.

## 1. Introduction

Since the introduction of DNA sequencing technologies and genomic diagnostics, increasing insights have been given into the evolution and variability of genomes. Over the past few decades, changes in the nucleotide composition and structure have been observed and extensively studied in a variety of organisms. A common observation when comparing genomes over time, across individuals or in different cell types is alterations of a single or multiple nucleotides. In many studies, observations of these alterations, termed genomic variants, have been associated with diseases, evolutionary processes and biodiversity [1,2]. The perceived benefits of a comprehensive understanding of genomic variants have driven immense efforts to collect and analyze genomic data. A range of bioinformatics software programs have been released to accurately quantify and describe the characteristics of genomic variants in a single or multiple genomes [3]. For such a software product to prevail over time, it is often subject to long-term update cycles, compared to novel competing software in the field and analyzed for technical errors by the community. These challenges of continuous software development require robust testing and the evaluation of results [4]. In the context of genomic variant detection, bioinformatics software needs to perform well under a variety of technical setups and the set of reported genomic variants should comply with a known ground truth.

Several existing applications provide functionalities for the performance of individual steps towards the end-to-end testing of genomic variant detection. Mason [5] and ART [6] are applications used to generate synthetic sequencing data for different sequencing technologies. PICARD [7] and BCFtools [8] are established tool suites for the manipulation of high-throughput sequencing data and variant call format (VCF) files. Krusche et al. [9] proposed a framework describing best practices for the benchmarking of small germline variants, which led to the highly recognized precisionFDA challenge [10]. The same authors released the widely community-accepted *hap.py* tools for variant set comparison. This tool suite implements elaborate methods to address complex cases where representational differences between sets of variants cannot be trivially fixed. A commonly used alternative to *hap.py* is RTG Tools (https://github.com/RealTimeGenomics/rtg-tools, accessed on 3 September 2024). Vcfdist [11] is another evaluation tool with a focus on using local phasing information. The recent NCbench [12] is an elaborate benchmark platform for sets of small genomic variants. It has a competitive advantage in interactivity and visualization. However, none of these individual applications offers an all-in-one solution for flexible, on-demand and easy-to-install end-to-end testing in genomic variant detection.

To this end, we introduce *CIEVaD* (an abbreviation for Continuous Integration and Evaluation of Variant Detection), a lightweight workflow collection designed for the rapid generation of synthetic test data from haploid reference genomes and the validation of software for genomic variant detection. CIEVaD is implemented in Nextflow [13], a workflow management framework for streamlined and reproducible data processing. The use of Nextflow additionally avoids manual installation efforts for the user via the deployment of software packages. CIEVaD is open-source and freely available on Github at https://github.com/rki-mf1/cievad (accessed on 3 September 2024).

## 2. Materials and Methods

The CIEVaD workflow collection contains two main workflows. The haplotype workflow generates synthetic data, whereas the evaluation workflow examines the accordance between sets of genomic variants. In the following, the term *synthetic* is used synonymously for in silico-generated data.

### 2.1. Haplotype Workflow

The haplotype workflow provides a framework for data generation. Synthetic data can be used for genomic variant calling, and the data generation scales computationally to many individuals. Moreover, different types of sequencing data can be specified. To begin with data generation, the one input parameter strictly required by the user is a reference genome. With only a given reference genome, the haplotype workflow generates a new haplotype sequence of the entire reference genome, a set of synthetic genomic variants (henceforth referred to as a *truthset*) and synthetic genomic reads. The truthset comprises single-nucleotide variants (SNVs) and short insertions and deletions (indels) of, at most, 20 nucleotides. The maximum length of indels, as well as the frequency of both variant types, can be adjusted via the workflow parameters. Since the variants of the truthset are homozygous, the alternative–allele ratio for each variant is defined as 1−ϵ, where ϵ is drawn from the read-specific error distribution.

In the initial step, the workflow indexes the given reference genome. Next, CIEVaD uses the Mason variator [5] for a given number *n* of individuals (the default is n=3). The result of this step is a haplotype sequence and truthset per individual. Here, a haplotype sequence is a copy of the reference genome with small genomic variants of the truthset inserted at pseudo-random locations. In other terms, the reference genome and a haplotype sequence differ in the variants of the corresponding truthset. The final step is the generation of synthetic genomic reads. Depending on the specified type of sequencing data (the default is *NGS*), either the Mason simulator generates pairs of genomic reads or PBSIM3 [14] generates a set of synthetic long reads. The read simulation parameters can be tuned via CIEVaD’s command-line interface or via a configuration file. In both cases, the workflow returns the alignment of the reads to the reference genome, e.g., for the manual inspection of the genomic variants and sequencing artifacts in the reads. Note that the data of each individual are computed in asynchronous parallel Nextflow processes, which scale effortlessly with additional CPU threads or compute nodes.

### 2.2. Evaluation Workflow

The objective of the evaluation workflow is to assess how successfully a third-party tool or workflow detects genomic variants. To assess the detection, CIEVaD’s evaluation workflow compares the set of genomic variants generated by the third-party tool or workflow (such a set is further referred to as a *callset*) with the corresponding truthset. The only strictly required input for the evaluation workflow is a set of callsets in the variant call format (VCF), either given as a folder path or a sample sheet. The outputs are reports about the accordance between corresponding truth- and callsets.

The evaluation workflow consists of only two consecutive steps. First, the open-source tool suite *hap.py* by Illumina Inc. is used to compare all truthsets with their corresponding callsets. In fact, its included submodule *som.py* identifies the number of correctly detected (TP), missed (FN), and erroneously detected (FP) variants. Due to using *som.py*, this comparison of the variants neglects the genotype information and only checks their positions in the genome, as well as the nucleotide composition. This default behavior is chosen since CIEVaD was initially developed for haploid pathogens where the presence of a variant itself reflects the genotype. Using the TP, FP, and FN counts, *som.py* reports the precision, recall, and F1-score of the variant detection process that yields the callset. The second step of the evaluation workflow is the computation of the average scores across the statistics of all individuals.

## 3. Results

**Figure A1:**
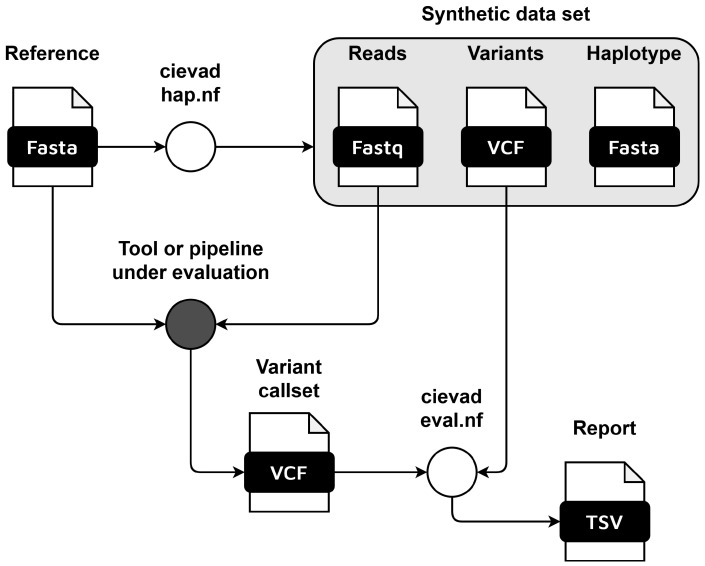
General overview of an end-to-end test using CIEVaD. The document pictograms show the data and file formats that are passed throughout the testing process. CIEVaD can be deployed around any variant detection software or pipeline as long as it returns a variant callset in variant call format (VCF).

To demonstrate the utility of CIEVaD, we implemented end-to-end tests (Appendix A Figure A1) for different variant detection software programs as part of their continuous integration frameworks.

### 3.1. Assessing Variant Detection from NGS Data as Part of SARS-CoV-2 Genome Reconstruction

We deployed CIEVaD to benchmark the variant detection routine within CoVpipe2 [15]. CoVpipe2 is a computational workflow for the reconstruction of SARS-CoV-2 genomes. One sub-process of CoVpipe2 applies the FreeBayes [16] variant detection method to a set of reference-aligned NGS reads. We implemented a test as part of CoVpipe2’s Github Actions (https://github.com/rki-mf1/CoVpipe2/actions/workflows/VariantCalling.yml, accessed on 3 September 2024) with the objective of obtaining F1-scores for the detection of SNVs and indels. The test consists of seven principal steps:

1. Install Conda and Nextflow;
2. Download a reference genome;
3. Run CIEVaD hap.nf;
4. Run CoVpipe2;
5. Prepare input for CIEVaD eval.nf;
6. Run CIEVaD eval.nf;
7. Check results.

In brief, the test runs the CIEVaD haplotype workflow with the default parameters, CoVpipe2 with the generated synthetic NGS data, and the CIEVaD evaluation workflow with the filtered callsets from CoVpipe2 and finally checks whether the F1-scores of the SNV and indel detection have decreased compared to the previous test scores. This rapid deployment of CIEVaD (v0.4.1) benchmarks the variant callsets of CoVpipe2 (v0.5.2) with F1-scores of 0.97 and 0.91 for SNVs and indels, respectively. The full table of results for this evaluation is given in Appendix B Table A1.

### 3.2. Assessing Variant Detection from Long-Read Data as Part of Nanopore Sequencing Data Analysis

**Figure A2:**
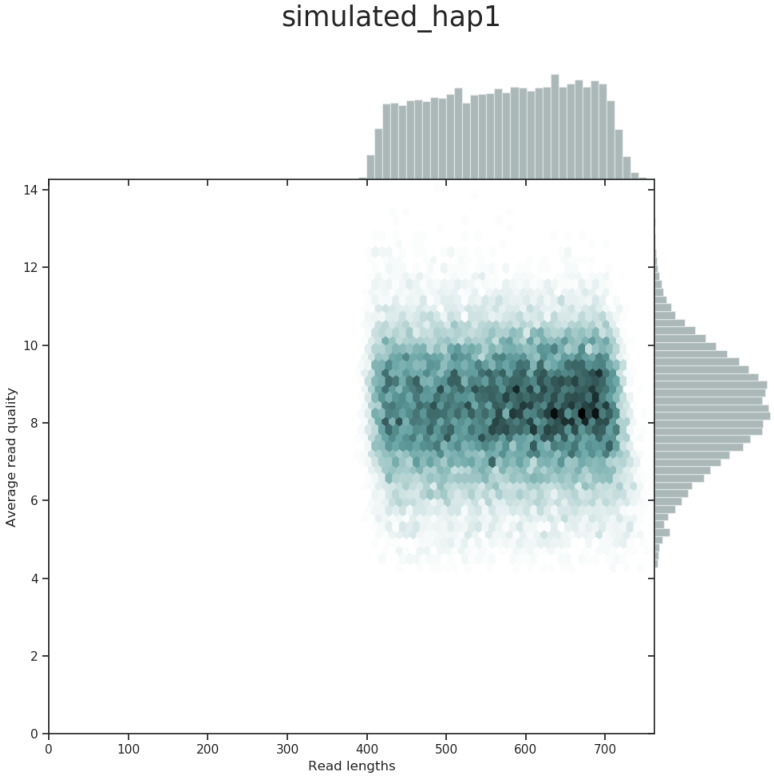
Heatmap of the length versus average quality of the synthetic long reads. The visualization is automatically generated with Nanoplot [20] as part of the poreCov workflow. The long reads were generated with PBSIM3 [14]. The length interval of the generated reads is chosen according to the read filter criteria of poreCov. The average read quality provides a challenging yet manageable signal-to-noise ratio for testing.

In order to deploy CIEVaD for a different genomic data type, we generated long reads with hap.nf using the *–read_type* parameter (see Supplementary Material A). The read type parameter invokes additional default parameters of the haplotype workflow that are tailored to a high-coverage long-read WGS experiment on the SARS-CoV-2 genome. With this setup, the haplotype workflow and its internal long-read module [14] generate a dataset with an average of 500-fold read coverage, per base accuracy of approximately 90% (Appendix C Figure A2), and an error distribution model trained on genomic data from Oxford Nanopore Technologies. We used the synthetic long-read dataset and the ground truth variants to test the variant detection of the poreCov [17] data analysis pipeline. Supplementary Material A shows how we adjusted poreCov (v1.9.4) to process the synthetic long-read dataset and, subsequently, how the evaluation workflow of CIEVaD verified poreCov’s results. Our evaluation of poreCov (Appendix D Table A2) shows F1-scores of 0.95 and 0.73 for SNVs and indels, respectively.

## 4. Conclusions

We introduce CIEVaD, an easy-to-apply tool suite used to assess small variant detection from short- and long-read datasets. CIEVaD is modular, extensible, and scaleable; requires no manual installation of internal software; and operates entirely on standard bioinformatics file formats. We show that the workflows of CIEVaD enable the rapid deployment of end-to-end tests, including the generation of synthetic genomic data and the evaluation of results from third-party variant detection software. Our tool greatly benefits bioinformaticians and data scientists in genomics by reducing the time spent on routine tasks for the evaluation and reporting of variant detection.

With the workflow design, open-source policy, file formats, software packaging, and documentation, we aim to comply with the PHA4GE best practices for public health pipelines (https://github.com/pha4ge/public-health-pipeline-best-practices/blob/main/docs/pipeline-best-practices.md, accessed on 3 September 2024). Within the scope of this work, we did not implement advanced continuous integration strategies for third-party software, but it is up to the user to apply arithmetic operations or use test frameworks around the results of CIEVaD. It should be mentioned here that CIEVaD is not restricted to synthetic data; the evaluation workflow also works with curated variant callsets from real sequencing data. Moreover, CIEVaD evaluates all variants reported within the VCF file, irrespective of their allele ratios (e.g., low-abundance variants in cases of intrahost variation). For the generation of synthetic read data, we chose to integrate the Mason and PBSIM3 read simulators due to their computational performance, maintenance status, and availability. The current limitations of CIEVaD comprise the ploidy of the provided reference genome and the types of variants. As the project and workflows were initially intended for viral pathogens, we have not implemented an option to generate diploid genomes and, hence, heterozygous variants. For the same reason, we have not provided an option to generate synthetic structural variants. In addition, structural variants require more sophisticated algorithms for variant set comparison. The features for heterozygous and structural variants remain subject to the users’ demands. Finally, although CIEVaD and its utility were demonstrated solely on software for SARS-CoV-2 data, there is no technical limitation regarding the usage of CIEVaD for SARS-CoV-2. We expect CIEVaD to work seamlessly with workflows tailored to other pathogens, e.g., RSVpipe [18], and even workflows that are not pathogen-specific, e.g., viralrecon (10.5281/zenodo.3901628) from the nf-core framework [19].

## Data Availability

The genomic read sequences used in this study were artificially (in silico) generated from the presented tools. The corresponding reference genome can be accessed from the NIH NCBI website via accession number NC_045512.2.

## Supplementary Materials

The following supporting information can be downloaded at: https://www.mdpi.com/article/10.3390/v16091444/s1.

## Appendix B

**Table A1:** Results of the CIEVaD evaluation workflow (v0.4.1) using the variant callsets of CoVpipe2 (v0.5.2). All values are average values across three synthetic individuals. The total in truthset, total in query, TP, FP, and FN are the averages of the individual case counts, hence from $R_{0}^{+}$. The precision, recall, and F1-score are floating point numbers from the interval [0,1]. Here, the term *query* is synonymous for callset, as used by the hap.py tools suite. The F1-score is the harmonic mean of the precision and recall.

Type	Total in Truthset	Total in Query	TP	FP	FN	Recall	Precision	F1-Score
indels	158.67	134.33	133.67	0.67	25	0.84	0.996	0.91
SNVs	287.33	268.67	268.67	0	18.67	0.93	1	0.97

## Appendix D

**Table A2:** Results of the CIEVaD evaluation workflow (v0.4.1) using the variant callsets of poreCov (v1.9.4). All values are average values across three synthetic individuals. The total in truthset, total in query, TP, FP, and FN are the averages of the individual case counts, hence from $R_{0}^{+}$. The precision, recall, and F1-score are floating point numbers from the interval [0,1]. Here, the term *query* is synonymous for callset, as used by the hap.py tools suite. The F1-score is the harmonic mean of the precision and recall.

Type	Total in Truthset	Total in Query	TP	FP	FN	Recall	Precision	F1-Score
indels	158.67	166	119	47	39.67	0.75	0.72	0.73
SNVs	287.33	263.33	261	2.33	26.33	0.91	0.99	0.95
