# Supplementary material for: CIEVaD: A Lightweight Workflow Collection for the Rapid and On-Demand Deployment of End-to-End Testing for Genomic Variant Detection
Source: Viruses. 2024 Sep 11;16(9):1444. doi: 10.3390/v16091444 (PMC11437481; doi:10.3390/v16091444)
Supplement: Supplementary file 1 [file viruses-16-01444-s001.zip › viruses-3156341-supplementary.pdf]

Supplemental material for "CIEVaD: a lightweight workflow collection for rapid and on demand deployment of end-to-end testing of genomic variant detection"

Thomas Krannich[1], **Dimitri Ternovoj**[1], **Sofia Paraskevopoulou**[1], **and Stephan Fuchs**[1]

[1]Genome Competence Center, Robert Koch Institute, Nordufer 20, 13353 Berlin, Germany

A. Shell commands used to test poreCov using CIEVaD. These shell commands were executed on a Linux compute node with an Ubuntu 20.04.6 LTS operating system and equipped with an AMD EPYC 9534 64-Core Processor. The working environment has the *conda* package management system and *singularity* container virtualization software pre-installed.

```
#!/bin/bash
#run synthetic data generation
conda create -n nextflow -c bioconda nextflow=23.10.1
conda activate nextflow
git clone https://github.com/rki-mf1/cievad.git && cd cievad
nextflow run hap.nf -profile local,conda --read_type ont
conda deactivate

#run poreCov
conda create -n nextflow-2104 -c bioconda nextflow=21.04.0
conda activate nextflow-2104
nextflow run replikation/poreCov -r 1.9.4 -dsl2 \
    --fastq "results/simulated_hap*.fastq" \
    -profile local,singularity --cores 2 --max_cores 8
conda deactivate

#run callset evaluation
echo "index,truthset,callset" > results/sample_sheet.csv
for i in {1..3}
do
    echo "${i},${PWD}/results/simulated_hap${i}.vcf,\
    ${PWD}/results/3.Lineages_Clades_Mutations/simulated_hap${i}\
    /SNP_simulated_hap${i}.pass.vcf" >> results/sample_sheet.csv
done
conda activate nextflow
nextflow run eval.nf -profile local,conda --sample_sheet results/sample_sheet.csv
column -s ',' -t results/summary.sompy.stats.csv | less -S
```
